# Redox Profile, Inflammatory and Cardiac Autonomic Nervous Activity of Children and Adolescents With Cystic Fibrosis

**DOI:** 10.1111/ped.70448

**Published:** 2026-06-21

**Authors:** Ana Carla Lima de França, Mateus Duarte Ribeiro, Ana Carolina Freitas Meireles, Lydiane Tavares Toscano, Larissa Araújo Maia, Constantino Giovanni Braga Cartaxo, Gilvan da Cruz Barbosa Araújo, Maria da Conceição Rodrigues Gonçalves, José Luiz de Brito Alves, Alexandre Sérgio Silva

**Affiliations:** ^1^ Department of Nutrition. Programa de Pós‐Graduação em Ciências da Nutrição Universidade Federal da Paraíba João Pessoa Brazil; ^2^ Laboratory of Physical Training Studies Applied to Performance and Health João Pessoa Brazil; ^3^ Department of Physical Education Programa Associado de Pós‐Graduação em Educação Física Universidade de Pernambuco/Universidade Federal da Paraíba João Pessoa Brazil; ^4^ Universidade Federal da Paraíba João Pessoa Brazil

**Keywords:** cardiac autonomic nervous activity, cystic fibrosis, oxidative stress, systemic inflammation

## Abstract

**Background:**

Cystic fibrosis (CF) is a genetic disease in which oxidative stress, chronic inflammation, and autonomic imbalance drive the pathophysiology; however, their interplay in children and adolescents with CF remains unclear. This study aimed to compare the cardiometabolic profile of children and adolescents with CF and healthy controls, and to investigate associations between heart rate variability (HRV), Forced Expiratory Volume in the first second (FEV_1_), oxidative stress, and inflammation.

**Methods:**

This cross‐sectional study included 12 patients with CF and 12 healthy controls (5–18 years). Were analyzed for oxidative stress markers (total antioxidant capacity‐CAOx, malondialdehyde‐MDA), and inflammatory (hs‐CRP, IL‐2, IL‐4, IL‐6, IL‐10, IL‐17A, IFN‐γ, and TNF‐α). HRV was assessed using a Bluetooth heart rate monitor.

**Results:**

Mean age was similar between groups (CF: 11.7 ± 4.5 years vs. CONT: 12.5 ± 4.2 years, *p* = 0.68). Mean FEV_1_ in the CF group was 78.7%. CF group, MDA was significantly elevated (CF: 3.8 ± 1.4 μM vs. CONT: 2.8 ± 0.3 μM, *p* = 0.03), while CAOx was lower (CF: 33.0% ± 4.3% vs. CONT: 45.3% ± 13.6%, *p* = 0.01); CRP (CF: 4.6 ± 5.67 mg/L vs. CONT: 0.6 ± 0.7 mg/L, *p* = 0.03), IL‐6 (CF: 53.0 ± 47.37 pg/mL vs. CONT: 25.1 ± 43.8 pg/mL, *p* = 0.02), and IL‐17 (CF: 89.3 ± 53.37 pg/mL vs. CONT: 41.4 ± 42.5 pg/mL, *p* = 0.03) were significantly elevated; Time‐domain variables were lower: SDNN (CF: 52 ± 24 ms vs. CONT: 88 ± 31 ms, *p* = 0.04), RMSSD (CF: 41 ± 21 ms vs. CONT: 68 ± 34 ms, *p* = 0.02), pNN50 (CF: 20% ± 16.3% vs. CONT: 35% ± 19.1%, *p* = 0.03); in the frequency domain, only low frequency (LF) was lower (CF: 1061 ± 1335 ms^2^ vs. CONT: 2979 ± 4896 ms^2^, *p* = 0.04).

**Conclusion:**

Children and adolescents with CF exhibit increased oxidative stress, systemic inflammation, and reduced sympathetic and parasympathetic activity, as well as associations among lung function, HRV, and antioxidant capacity.

**Abbreviations**
BMI: body mass index
CAOx: total antioxidant capacity
CBA: cytometric bead array
CF: cystic fibrosis
CFTR: cystic fibrosis transmembrane regulator gene
CHO: carbohydrate
CONT: control
CRP: C‐reactive protein
EBC: exhaled breath condensate
FEV_1_
: forced expiratory volume in 1 s
FVC: forced vital capacity
HF: high frequency
HRV: variability of heart rate
IL: interleukin
LF: low frequency
LIP: lipids
MDA: malondialdehyde
pNN50: number of pairs of adjacent RR intervals that differ by more than 50 ms during recording
PTN: protein
RDNN: standard deviation of all RR intervals
rMSSD: square root of the mean of the sum of squares of the
TNF‐α: tumor necrosis factor alpha
VET: total energy value
VIT: vitamins

## 1 Introduction

Cystic fibrosis (CF), also known as mucoviscidosis, is an autosomal recessive genetic disorder caused by mutations in the cystic fibrosis transmembrane conductance regulator (CFTR) gene, located on the long arm of chromosome 7, which encodes the CFTR protein [1]. More than one thousand mutations have been identified in the CFTR gene, and genotype–phenotype correlations have been described, with clinical manifestations ranging from mild to severe [2].

The genetic defect impairs chloride secretion, leading to intracellular electronegativity and enhanced sodium influx. This imbalance promotes the production of viscous mucus in secretory organs, particularly within the respiratory system, as well as in the digestive and reproductive tracts, causing organ dysfunction [3]. The most severe pathological consequences occur in the lungs, where chronic hyperinflammation leads to airway wall destruction and fibrosis, ultimately resulting in progressive lung function decline, the leading cause of mortality in individuals with CF [4, 5].

Among the pathological mechanisms of CF, redox imbalance in epithelial cells and extracellular fluids leads to abnormal generation of reactive oxygen species. Consequently, oxidative stress has been implicated as a contributing factor in disease pathophysiology, as supported by systematic reviews and meta‐analyses [6, 7]. Galli et al. [6] reported that patients with impaired lung function exhibited significantly higher plasma levels of lipid hydroperoxides. Causer et al. [7] demonstrated increased oxidative stress markers (protein carbonyls, 8‐iso‐prostaglandin F2α, and malondialdehyde) in clinically stable individuals with CF, along with reduced antioxidant capacity.

In addition to oxidative processes, a pronounced inflammatory response is also observed in patients with CF. A systematic review by Cantin et al. [8] identified multiple inflammatory mechanisms, including dysregulation of innate and adaptive immunity, alterations in cell membrane lipid composition, defects in transcription factor signaling, and abnormal kinase responses. Pulmonary inflammation in CF is predominantly neutrophilic, with activated neutrophils releasing oxidants and proteases that contribute to progressive lung damage. Consequently, patients with CF exhibit significantly higher levels of interleukin‐6 (IL‐6), C‐reactive protein (CRP), and tumor necrosis factor‐α (TNF‐α) compared with healthy individuals [9].

Similar to oxidative stress and inflammation, sympathetic hyperactivity is considered an important component of the pathophysiology of several chronic diseases, including diabetes [10], cancer [11], hypertension [12], and Alzheimer's disease [13]. It has been associated with increased mortality in adults and older populations [14].

However, to date, only five studies have investigated autonomic modulation in individuals with CF [15, 16, 17, 18, 19]. Although these studies employed relatively novel techniques, their findings are limited by methodological weaknesses. Florêncio et al. [16] reported greater sympathetic activity at rest in children with CF compared with healthy controls, which persisted after a six‐minute walk test. However, their analysis was restricted to frequency‐domain indices (low frequency [LF], high frequency [HF], and the low/high frequency ratio [LF/HF]), measures whose interpretation has been questioned because they may reflect influences beyond autonomic modulation.

McNarry and Mackintosh [18] demonstrated that both time‐ and frequency‐domain heart rate variability parameters show good to excellent reproducibility in children with CF. The remaining studies report inconsistent findings. De Carvalho et al. [15] identified an association between CF, colonization by *Pseudomonas aeruginosa* , and increased sympathetic activity. Lugão et al. [17], who evaluated only individuals with CF, found significant associations between frequency‐domain indices (LF and HF), nocturnal hypoxemia, obstructive sleep apnea syndrome, and reduced lung function; however, the absence of a control group represents an important limitation. In contrast, a study in adults with CF and severe pulmonary impairment reported preserved autonomic function compared with healthy controls [19].

Although inflammation, oxidative stress, and sympathetic hyperactivity are recognized contributors to the pathophysiology of chronic diseases, none of these previous studies have examined the association among these three variables simultaneously in the same group of patients with CF. Therefore, this study aims to characterize the redox, inflammatory, and cardiac autonomic profiles of children and adolescents with CF and to investigate the associations between heart rate variability (HRV), lung function (FEV_1_), oxidative stress, and inflammation.

## 2 Methods

### 2.1 Subjects

This observational cross‐sectional study included children and adolescents aged 5 to 18 years with a diagnosis of CF treated at a university hospital. Healthy controls were recruited from students living in nearby communities. Follow‐up and data collection were conducted between August 2023 and March 2024.

Sample size was calculated according to Eng [20] using Gpower 3.1 software (Franz Faul, University of Kiel, Germany). Estimates were based on the cross‐sectional study by Florêncio et al. [16], which reported greater sympathetic activity at rest in children with CF compared with healthy controls (LF: CF: 53.2 ± 15.0; Healthy: 32.8 ± 7.9; *p* = 0.0003), corresponding to an effect size of 1.7. Assuming α = 0.05 and a statistical power of 80%, the minimum required sample size was 12 participants per group.

Inclusion criteria for CF patients were: confirmed diagnosis of CF based on a positive sweat test and/or identification of CFTR mutations; no indication for or prior lung transplantation; absence of renal or hepatic dysfunction; and clinical stability, defined as no hospitalization or pulmonary exacerbation within 30 days before assessment. For the healthy group, inclusion criteria were: no family history of CF or other chronic disease; no use of anti‐inflammatory drugs or antibiotics within 30 days; no regular consumption of red grape juice, dietary supplements, vitamins, or grape‐derived polyphenol products; and participation in competitive sports or regular structured physical training. Participants who failed to complete at least one assessment (blood collection or measurement of cardiac autonomic activity) were excluded.

Written informed consent was obtained from the parents or legal guardians of all participating children and adolescents. In addition, written informed assent was obtained from literate children and adolescents, in accordance with Resolution No 466, of December 12, 2012, of the National Health Council [21]. The study was approved by the Research Ethics Committee of Lauro Wanderley University Hospital/UFPB under protocol number 5.481.257.

### 2.2 Experimental Design

**FIGURE 1:**
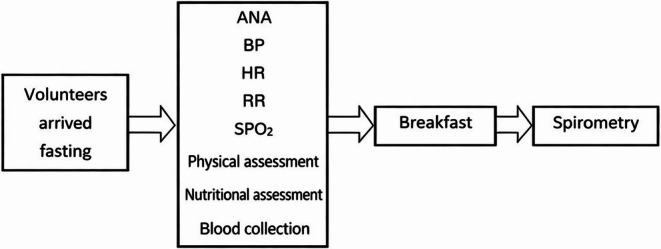
Case–control study design to characterize the sample studied. ANA, autonomic nervous activity; BP, blood pressure; HR, heart rate; RR, respiratory rate; SpO2, oxygen saturation.

Initially, a semi‐structured questionnaire specifically developed for this study was administered to collect sociodemographic and clinical data. Subsequently, participants underwent clinical and nutritional assessments. Information on the genetic diagnosis of cystic fibrosis was obtained from medical records, which identified the F508DEL CFTR mutation. Blood samples were collected to assess redox and inflammatory profiles, and cardiac autonomic nervous system activity and pulmonary function were also evaluated (Figure 1).

### 2.3 Anthropometric Assessment

Anthropometric measurements were obtained to evaluate nutritional status using body mass index (BMI) for age, classified according to the WHO 2007 growth reference for individuals older than 5 years [22]. Body weight was measured using a digital scale (Plenna Lumina, model MEA‐02550, Brazil; precision 0.1 kg; capacity 150 kg). Height was measured using a portable stadiometer (Sanny Standard, São Paulo, Brazil; precision of 0.1 cm). Participants stood upright, barefoot, without headwear, with arms at their sides, and looking straight ahead during measurement [23].

### 2.4 Assessment of Lung Function

Pulmonary function was assessed by spirometry using a portable Contec Sp10w device (Contec Medical Systems, Hebei, China). Three maneuvers were performed, and the highest values of Forced Vital Capacity (FVC) and Forced Expiratory Volume in the first second (FEV_1_) were selected. The following parameters were evaluated: FVC, FEV_1_, peak expiratory flow (PEF), forced expiratory flow between 25% and 75% of FVC (FEF_25%‐75%_), and Tiffeneau index (FEV_1_/FVC), following established protocols [24]. Participants were instructed to avoid vigorous physical activity for 24 h before the test [24]. Results are expressed as absolute values and as percentages of predicted values according to sex, age, and height. Forced FEV_1_ was classified according to severity in patients with mild (FEV_1_ ≥ 70% of predicted), moderate (FEV_1_ 60%–69% of predicted) and, severe (FEV_1_ 50%–59% of predicted) lung disease [25].

### 2.5 Blood Collection and Biochemical Analysis

Ten milliliters of venous blood were collected. The samples were then centrifuged at 3000 rpm for 10 min to obtain serum or plasma, aliquoted into microtubes, and stored at −20°C until analysis. Trained nurses consistently performed blood collection in a laboratory with appropriate infrastructure.

Oxidative stress: Antioxidant activity was assessed by total antioxidant capacity (CAOx), determined in plasma by measuring the scavenging activity of the free radical 2,2‐diphenyl‐1‐picrylhydrazyl (DPPH) [26]. Lipid peroxidation was quantified by malondialdehyde (MDA) through the reaction of thiobarbituric acid with the decomposition products of hydroperoxides [27].

Inflammation: Plasma high‐sensitivity C‐reactive protein (hs‐CRP) concentrations were quantified by immunoturbidimetry using serum samples and a commercial assay kit (Labtest, Belo Horizonte, Brazil), following the manufacturer's instructions. For hs‐CRP calibration, the Calibra Plus PCR‐ultra calibrator (Labtest: Ref‐345) was used. Absorbance was obtained on the Labmax 240 premium automatic analyzer (Labtest, Belo Horizonte, Brazil) at a wavelength of 540 nm. Systemic cytokine levels (IL‐2, IL‐4, IL‐6, IL‐10, IL‐17A, IFN‐γ and, TNF‐α) were determined via Cytometric Bead Array (CBA) kit (Becton Dickinson (BD) Biosciences, San Jose, CA, USA) as previously described [28].

### 2.6 Heart Rate Variability

Cardiac autonomic activity was assessed using heart rate variability (HRV) from R–R interval recordings obtained with an Android smartphone with the ELITE HRV application (Asheville, NC, USA). Participants wore an elastic chest strap equipped and a Bluetooth heart rate transmitter (Polar H10, Polar Electro Oy, Kempele, Finland). This instrument was validated against electrocardiogram recording at rest and during exercise [29].

Artifact‐free R‐R intervals were recorded for 5 min. When artifacts were detected, recordings were repeated. The volunteers remained seated for 5 min, refraining from speaking or moving. Before starting the recording, a 1 min period was established for heart rate stabilization [30]. HRV parameters were analyzed in the time domain (mean and standard deviation of the R‐R intervals) and in the frequency domain (low‐ and high‐frequency intervals and the low/high frequency ratio).

### 2.7 Statistical Analysis

Data are presented as mean ± standard deviation (SD). Normality of data distribution was assessed using the Shapiro–Wilk test, and homogeneity of variances was evaluated using Levene's test. For normally distributed variables, between‐group comparisons were performed using the independent samples *t*‐test. For non‐normally distributed variables, the Mann–Whitney *U* test was applied.

Effect size was calculated using Cohen's *d* and interpreted as follows: Small effect: *d* = 0.2 (the difference between the groups is trivial, even if statistically significant); Moderate effect: *d* = 0.5 (the difference between the groups is noticeable); Large effect: *d* = 0.8 (the difference between the groups is substantial) [31]. Spearman's rank correlation coefficient was used to examine associations among heart rate variability (HRV) indices, pulmonary function parameters, inflammatory markers, and oxidative stress markers. Correlation strength was classified as follows: < 0.3 negligible; > 0.3 to 0.5 weak; > 0.5 to 0.7 moderate; > 0.7 to 0.9 strong; > 0.9 very strong; 1.0 perfect [32]. A confidence level of 5% (*p* < 0.05) was adopted for all tests. Jamovi Statistics software, Version 2.3.26 for Windows, was used for analysis.

## 3 Results

The characteristics of the study participants are presented in Table 1. The sample consisted of 12 children and adolescents with CF and 12 healthy controls with a similar mean age. According to medical records, 45% of patients with CF carried the F508del mutation (ΔF508) at position 508 in the transmembrane conductance regulator protein. A total of 30% (*n* = 4) of the CF patients had low BMI for their age, while the others had adequate BMI; in the control group, all were eutrophic. Patients with CF showed significantly lower FEV_1_, FVC, peak expiratory flow (PEF). They forced expiratory flow between 25% and 75% of FVC (FEF_25%‐75%_) compared with controls, and according to Cystic Fibrosis Foundation (CFF) criteria, they were classified as having mild lung disease (FEV_1_ ≥ 70% of predicted) [25]. The mean SpO2 was 97% in the CF group and 98% in the CONT group (*p* = 0.08).

**TABLE 1:** Characterization of the study sample in patients with Cystic Fibrosis and control group in Joao Pessoa, Brazil.

	CF (*n* = 12)	CONT (*n* = 12)	*p*
*Demographics*			
Masculine, *n* (%)	7 (45)	6 (50)	—
Age (years)	11.7 ± 4.5	12.5 ± 4.2	0.68
*Anthropometric*			
Weight (kg)	28.9 ± 12.4	47.4 ± 17.4	0.00*
Height (cm)	137 ± 0.2	152 ± 0.1	0.06
BMI (kg/m^2^)	14.7 ± 2.3	19.4 ± 3.4	0.00**
Genotyping			
CFTR (com F508del) %	5 (41.6)	—	—
*Chronic airway colonization*			
*Pseudomonas aeruginosa*, *n* (%)	3 (25)	—	—
*Lung function*			
FEV_1_ (L)	2.2 ± 0.5	3.7 ± 0.3	0.00**
FEV_1_ (%)	78.7 ± 11.0	99.6 ± 5.6	0.00**
FVC (L)	2.3 ± 0.5	3.8 ± 0.4	0.00**
FVC (%)	75.3 ± 11.3	93.3 ± 4.6	0.00**
EFP (L)	3.4 ± 0.8	4.2 ± 0.7	0.03*
EFP (%)	64.5 ± 18.1	91.1 ± 3.0	0.00*
FEF_25%–75%_ (L/min)	3.1 ± 0.7	3.3 ± 0.6	0.23
FEF_25%–75%_ (%)	97.5 ± 20.1	113.1 ± 15.3	0.04*
FEV_1_/FVC	0.9 ± 0.0	0.9 ± 0.0	0.68
SpO2 (%)	97 ± 1.5	98 ± 0.8	0.08

*Note:* Data are presented as mean ± standard deviation.

Abbreviations: BMI, body mass index; CF, cystic fibrosis patient group; CFTR, cystic fibrosis transmembrane regulatory gene; CONT, control group; EFP, peak expiratory flow; FEF_25%‐75%_, forced expiratory flow between 25% and 75% of FVC; FEV1, forced expiratory volume in 1 s; FVC, forced vital capacity; L, liters; SpO2, peripheral oxyhemoglobin saturation.

* *p* < 0.05 (independent *t*‐test).

** *p* < 0.001 (independent *t*‐test).

Regarding dietary intake (Table 2), considering the recommendations for age according to the Dietary Reference Intakes [33], the volunteers, both in the CF and CONT groups, had diets with high carbohydrates and lipid intakes, with no difference between groups. Both groups had low intake of vitamins D and B9. The CF group also had low vitamin A intake. However, a statistically significant difference between groups was observed only for vitamin A; no significant differences were found for the other nutrients or micronutrients analyzed.

**TABLE 2:** Food consumption during the study in patients with cystic fibrosis and control group in João Pessoa, Brazil.

	CF	CONT	Recommendations^a^	*p*
VET (kcal)	2177 ± 0.322	2700 ± 0.348	1742–3152	0.00**
CHO (g)	312.4 ± 89.9	319.8 ± 107.7	100–130	0.85
PTN (g)	41.7 ± 14.3	52.2 ± 12.4	19–52	0.37
LIP (g)	76.0 ± 9.0	74.7 ± 8.2	25–35	0.72
VIT A/retinol (μg)	311.5 ± 247.7	414.0 ± 158.0	400–900	0.04**
VIT C/ascorbic acid (mg)	103.9 ± 129.9	115.1 ± 129.0	25–75	0.83
VIT D/calciferol (μg)	3.6 ± 1.6	4.5 ± 1.1	5.0	0.11
VIT E/α‐tocopherol (mg)	13.1 ± 8.8	15.1 ± 5.3	6–12	0.17
VIT B1/thiamine (mg)	1.2 ± 0.4	1.3 ± 0.4	0.6–1.2	0.60
VIT B2/riboflavin (mg)	1.3 ± 0.3	1.5 ± 0.4	0.6–1.3	0.43
VIT B3/niacin (mg)	16.2 ± 4.8	17.0 ± 6.2	8–16	0.73
VIT B6/pyridoxine (mg)	1.3 ± 0.3	1.6 ± 0.2	0.6–1.3	0.87
VIT B9/folic acid (μg)	186.2 ± 33.1	193.8 ± 47.9	200–400	0.65
VIT B12/cobalamin (μg)	3.5 ± 1.0	3.6 ± 1.2	1.2–2.4	0.79
Selenium (μg)	63.6 ± 15.6	66.7 ± 16.2	30–55	0.64
Zinc (mg)	11.5 ± 1.7	12.0 ± 1.1	5–11	0.41
Manganese (mg)	2.4 ± 0.7	2.8 ± 0.6	1.5–2.2	0.13

*Note:* Data are presented as mean ± standard deviation.

Abbreviations: CF, Cystic Fibrosis patient group; CHO, carbohydrate; CONT, control group; LIP, lipids; PTN, protein; VET, total energy value; VIT, vitamins.

^a^ Recommendations for children and adolescents according to the Dietary reference intakes guidelines (daily values), Padovani et al. [33].

** *p* < 0.001 (independent *t*‐test or Mann–Whitney test for nonparametric data).

**FIGURE 2:**
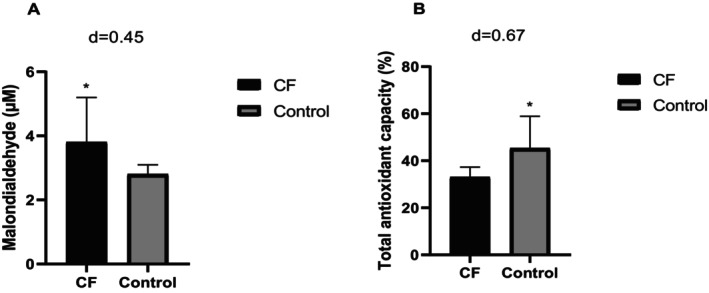
Comparison of MDA (A) and CAOx (B) analyses in children and adolescents with cystic fibrosis and the control group. Values represent mean ± standard deviation. Abbreviations: CAOx, total antioxidant capacity; CF, Cystic Fibrosis patient group; MDA, malondialdehyde. Independent *t*‐test, *p < 0.05.

Figure 2 shows the MDA results in the CF and control groups. The serum concentration of MDA was significantly elevated in the CF group, 35.7% higher compared to the control group (CF: 3.8 ± 1.4 μM vs. CONT: 2.8 ± 0.3 μM, *p* = 0.03, *d* = 0.45, small effect). Reinforcing the finding of greater oxidative stress, the CF group presented a 27.1% lower concentration of CAOx (CF: 33.0% ± 4.3% vs. CONT: 45.3% ± 13.6%, *p* = 0.01, *d* = 0.67, moderate effect).

**FIGURE 3:**
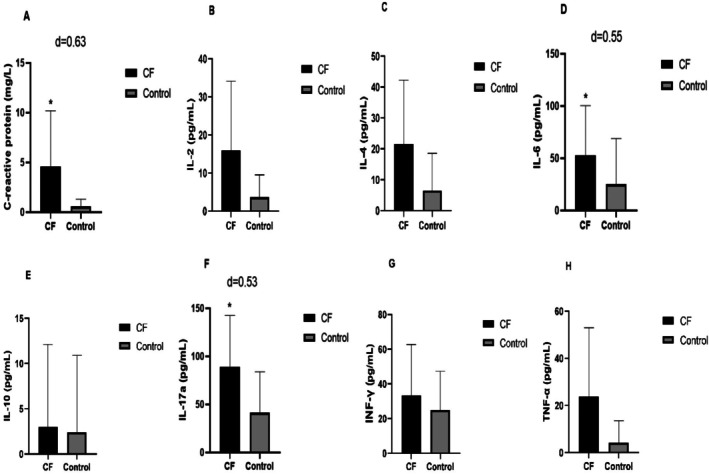
Comparison of analyses of inflammation markers in children and adolescents with Cystic Fibrosis and control group. Abbreviations: CF, Cystic Fibrosis patient group; CRP, C‐reactive protein (A); interleukin (IL): IL‐2 (B), IL‐4 (C), IL‐6 (D), IL‐10 (E), IL‐17a (F); interferon gamma: IFN‐γ (G) and tumor necrosis factor‐α: TNF‐α (H). Values represent mean ± standard deviation. Independent *t*‐test or Mann Whitney test for nonparametric data, *p < 0.05.

In addition to greater oxidative stress, the data presented in Figure 3 show that the CF group exhibited significantly higher levels of inflammation markers compared to the control group: Mean hs‐CRP levels were approximately 6.7‐fold higher in CF patients (CF: 4.6 ± 5.6 mg/L vs. CONT: 0.6 ± 0.7 mg/L, *p* = 0.03, *d* = 0.63, moderate effect). Similarly, IL‐6 levels showed a 111.2% increase (CF: 53.0 ± 47.3 pg/mL vs. CONT: 25.1 ± 43.8 pg/mL, *p* = 0.02, *d* = 0.55, moderate effect), and IL‐17A concentrations were 115.7% higher (CF: 89.3 ± 53.3 pg/mL vs. CONT: 41.4 ± 42.5 pg/mL, *p* = 0.03, *d* = 0.53, moderate effect), compared to controls. No significant differences were observed for the remaining cytokines (IL‐2, IL‐4, IL‐10, IFN‐γ, TNF‐α).

**FIGURE 4:**
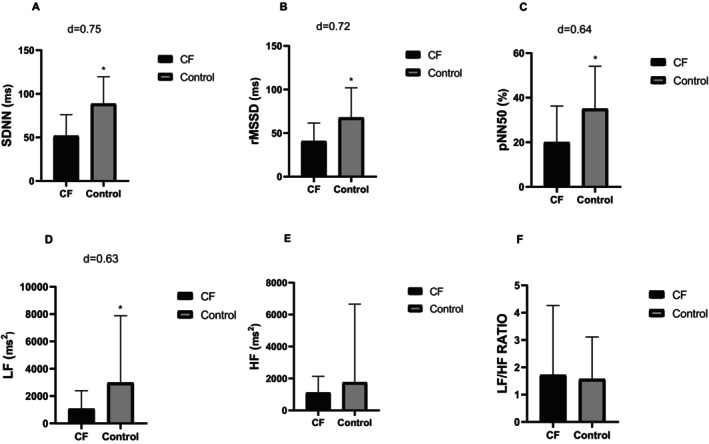
Comparison of HRV in children and adolescents with Cystic Fibrosis and the control group. Abbreviations: CF, Cystic Fibrosis patient group; SDNN: Standard deviation of all RR intervals (A); rMSSD: Square root of the mean of the sum of squares of the differences between adjacent RR intervals (B); pNN50: Number of pairs of adjacent RR intervals that differ by more than 50 ms during recording (C); LF, low frequency (D); HF, high frequency (E); LF/HF ratio (F). Values represent mean ± standard deviation. Independent *t*‐test or Mann Whitney test for nonparametric data, *p < 0.05.

Figure 4 shows the HRV results in the CF and control groups. It can be observed that the time domain variables were statistically lower in the CF group: SDNN (CF: 52 ± 24 ms vs. CONT: 88 ± 31 ms, *p* = 0.04, *d* = 0.75, moderate effect), RMSSD (CF: 41 ± 21 ms vs. CONT: 68 ± 34 ms, *p* = 0.02, *d* = 0.72, moderate effect), and pNN50 (CF: 20% ± 16.3% vs. CONT: 35% ± 19.1%, *p* = 0.03, *d* = 0.64, moderate effect), being 41.5%, 40.8%, 43.3% lower in relation to the control group, respectively, indicating reduced parasympathetic activity among CF patients. In the frequency domain, LF was 64.3% lower in the CF group (CF: 1061 ± 1335 ms^2^ vs. CONT: 2980 ± 4896 ms^2^, *p* = 0.04, *d* = 0.63, moderate effect), which would indicate lower sympathetic activity in this group; however, for the LF/HF ratio (sympathovagal indicator), there was no statistical difference between the groups.

In addition to the differences observed between CF and healthy controls, a correlation was identified between heart rate variability (HRV) and pulmonary function (FEV_1_) (Table 3). FEV_1_ was positively correlated with SDNN (*r* = 0.43, *p* = 0.03) and RMSSD (r = 0.43, *p* = 0.04), although these correlations were of weak magnitude. No significant correlations were observed between FEV_1_ and pNN50 (*r* = 0.35, *p* = 0.09) or between FEV_1_ and the LF/HF ratio (*r* = 0.31, *p* = 0.13). Regarding markers of oxidative stress (CAOx and MDA) and inflammation (CRP, IL‐6, and IL‐17A), no significant associations with HRV were observed. However, a weak but significant positive correlation was detected between FEV_1_ and CAOx (*r* = 0.42, *p* = 0.04) (Table 4).

**TABLE 3:** Correlation between variables, heart rate variability, pulmonary function, oxidative stress markers, and inflammatory indicators.

*n* = 24	FEV_1_		MDA		CAOx		CRP		IL‐6		IL‐17A	
	Rho	*p*	Rho	*p*	Rho	*p*	Rho	*p*	Rho	*p*	Rho	*p*
SDNN	0.43	0.03*	−0.37	0.07	0.39	0.06	−0.36	0.07	−0.45	0.06	−0.34	0.11
rMSSD	0.43	0.04*	−0.29	0.16	0.39	0.06	−0.38	0.06	−0.37	0.08	**−**0.36	0.09
pNN50	0.35	0.09	−0.22	0.29	0.33	0.10	−0.41	0.08	−0.35	0.10	−0.36	0.09
LF	0.37	0.07	−0.27	0.18	0.27	0.06	−0.03	0.83	−0.49	0.03	−0.46	0.03
HF	0.23	0.27	−0.23	0.26	−0.02	0.87	0.00	0.98	−0.34	0.11	−0.33	0.12
LF/HF	0.31	0.13	−0.03	0.86	0.25	0.24	0.07	0.74	−0.18	0.40	−0.31	0.15

Abbreviations: CAOx, total antioxidant capacity; FEV_1_, forced expiratory volume in 1 s; HF, high frequency; LF, low frequency; MDA, malondialdehyde; pNN50, number of pairs of adjacent RR intervals that differ by more than 50 ms during recording; rMSSD, square root of the mean of the sum of squares of the differences between adjacent RR intervals; SDNN, standard deviation of all RR intervals.

* Spearman correlation test.

**TABLE 4:** Correlation between variables, pulmonary function, oxidative stress markers, and inflammatory indicators.

*n* = 24	FEV_1_	
	Rho	*p*
MDA	−0.08	0.70
CAOx	0.42	0.04*
CRP	−0.37	0.07
IL‐6	−0.30	0.17
IL‐17A	−0.24	0.27

Abbreviations: CAOx, total antioxidant capacity; CRP, C‐reactive protein; FEV_1_, Forced Expiratory Volume in the first second; interleukin (IL): IL‐6, IL‐17A; MDA, malondialdehyde.

* Spearman correlation test.

## 4 Discussion

This study found that children and adolescents with CF exhibit increased oxidative stress, significantly elevated levels of some inflammation markers (CRP, IL‐6, IL‐17A), and to reduced sympathetic and parasympathetic activity. However, significant correlations were observed between lung function (FEV_1_) and time‐domain variables (SDNN and rMSSD), as well as between FEV_1_ and CAOx.

The oxidative stress observed in the present study corroborates findings from previous investigations [34, 35, 36]. Dysfunction of the CFTR gene leads to redox imbalance in epithelial cells and extracellular fluids, resulting in abnormally high generation of reactive oxygen species [37]. Elevated plasma MDA levels have been consistently reported in CF patients [35], including across different biological matrices such as exhaled breath condensate, sputum, and plasma [34]. In contrast, reduced total antioxidant capacity has been reported in this population, reflecting impaired antioxidant defense mechanisms [36].

Although most studies report increased oxidative stress in cystic fibrosis, discrepant findings have also been described. Some investigations found no differences in MDA levels [36], antioxidant enzyme activity such as GPx and SOD [9, 35, 38], or total antioxidant capacity [9, 35, 38]. These inconsistencies may be partly explained by clinical stability at the time of assessment and by the routine use of antioxidant supplementation in CF management. In the present study, CF patients showed low intake of vitamins A, D, and B9, which may have contributed to the observed oxidative imbalance, as previously suggested by Oliveira et al. [9].

In addition to greater oxidative stress, the CF group showed significantly elevated levels of some markers of inflammation. Activation of the innate immune response is mediated by increased TNF‐α, which stimulates neutrophils and macrophages and enhances antigen presentation to the adaptive immune system. Therefore, chronic inflammation would be mainly modulated by TNF‐α, IFN‐γ, IL‐6, and IL‐8, with reduced levels of the other interleukins [39]. IL‐6 is an important cytokine involved in several immunological processes, especially in the synthesis of acute‐phase proteins by the liver, and in the metabolic regulation of CRP [40]. In fact, the present study demonstrated elevated levels of both IL‐6 and CRP.

Corroborating our findings, Oliveira et al. [9] and Bene et al. [41] reported higher CRP and IL‐6 levels in patients with CF, with positive associations between these markers and greater disease severity, assessed by FEV_1_. In contrast, although increased TNF‐α, IFN‐γ, IL‐4, and IL‐10 levels have been reported in previous studies [9, 39, 42], no differences were observed in the present study, possibly reflecting variations in disease status. Notably, IL‐17A levels were elevated, a finding rarely documented in pediatric CF but previously reported in sputum samples [43], with lower levels described in patients infected with *Pseudomonas aeruginosa* [42].

Another finding in this study was reduced sympathetic and parasympathetic activity in CF. Mutations in the CFTR gene can lead to abnormalities in the autonomic nervous system, resulting in changes in heart rate variability (HRV) [44]. However, autonomic activity is very sensitive to the physiological state, so that only the classic changes in CF, including oxidative stress and systemic inflammation, may be sufficient to provoke a response from the nervous system, altering the autonomic balance, as proposed by López‐Cano et al. [10], in which autonomic activity, oxidative stress, and inflammation system feedback on each other.

Previous studies have investigated autonomic dysfunction in CF [15, 16, 17, 19]. In the present study, autonomic dysfunction was characterized by reduced parasympathetic activity in the time domain, without evidence of increased sympathetic modulation in the frequency domain. Similar findings were reported by Florêncio et al. [16] in children with mild lung disease, whereas Lugão et al. [17] observed frequency‐domain alterations only in patients with severe impairment. In contrast, normal autonomic function has been described in adults with advanced disease [19], suggesting that autonomic responses may vary according to disease stage, although parasympathetic modulation is expected to decline as lung disease progresses.

Adverse clinical conditions may contribute to autonomic imbalance and are likely related to disease severity and progression. De Carvalho et al. [15] identified sympathetic hyperactivity in children with CF and poorer lung function in those colonized by *Pseudomonas aeruginosa* . In the present study, three patients were chronically colonized; although clinically stable and representing a small proportion of the sample, this condition may have partially influenced inflammatory and autonomic outcomes. Future studies with larger samples should stratify analyses according to colonization status. Considering the present findings, autonomic, inflammatory, and oxidative stress variables were altered in CF patients, suggesting interactions among these systems. Correlation analyses revealed weak but consistent associations between pulmonary function (mild lung disease) and HRV time‐domain indices (SDNN and rMSSD), but not frequency‐domain variables, reinforcing the relevance of parasympathetic modulation in CF.

The absence of correlation with frequency‐domain variables may be interpreted in light of Billman [45] findings which questioned the methodological validity of these measures as indicators of sympathetic and parasympathetic activity, as they may reflect physiological processes beyond autonomic modulation. LF and the LF/HF indices are commonly used to represent sympathetic activity; nevertheless, studies involving parasympathetic blockade have shown reductions of at least 50% in LF [45, 46], indicating a substantial parasympathetic contribution to this parameter.

A weak correlation was also observed between FEV_1_ and CAOx, suggesting that reduced pulmonary function is associated with diminished total antioxidant capacity. This finding is consistent with the vulnerability of the lungs to oxidative stress in CF and with impaired absorption of dietary antioxidants due to pancreatic insufficiency [47, 48]. However, these correlations should be interpreted with caution given the small sample size, and potential associations involving frequency‐domain HRV indices and inflammatory or oxidative markers cannot be excluded.

The main strength of this study is the simultaneous assessment of oxidative stress, inflammatory markers, and autonomic nervous activity in the same group of children and adolescents with cystic fibrosis. To our knowledge, no previous study has evaluated these three domains concurrently using a broad set of indicators. The limitations include limited assessment of oxidative stress, restricted to malondialdehyde (MDA) and total antioxidant capacity (CAOx), without evaluation of protein and DNA damage markers or specific antioxidant enzymes. Additionally, the relatively small sample size should be acknowledged. However, similar sample sizes have been reported in studies involving pediatric CF populations [16, 38], reflecting the disease's prevalence and clinical instability. In addition, the cross‐sectional design precludes evaluation of changes over the course of disease progression.

Even though this was a characterization study, the data point to practical and clinical implications: that oxidative stress should not be considered an absolute condition in CF, and that assessment of redox balance should account for nutritional status, particularly antioxidant vitamin intake. Although cytokines are specific markers of inflammation, their high cost limits routine use, whereas CRP, despite being nonspecific, is a low‐cost, practical indicator of the patient's current inflammatory state.

Finally, an important practical and clinical implication of this study is the notable reduction in patients' parasympathetic activity achieved through a technique that is cost‐effective, as it uses only a low‐cost heart rate transmitter belt. Smartphone applications have been validated, such as the electrocardiogram [25]. Thus, it tends to consolidate itself as the first domestic clinical marker of CF, as it can be used by CF patients themselves or their families with a small investment and a simple assessment technique. Therefore, we consider analyzing heart rate variability the most practical implication of this study.

## 5 Conclusion

Children and adolescents with CF exhibit higher oxidative stress, significantly elevated levels of certain inflammatory markers, and reduced sympathetic and parasympathetic activity, even among patients considered to have mild pulmonary disease, which represents the main finding compared to previous literature. However, a weak correlation was observed between lung function (mild pulmonary disease), HRV (SDNN and rMSSD), and CAOx. For future research, we suggest increasing the sample size to confirm this HRV response and to support additional findings.

## Author Contributions

A.C.L.F.: One of the creators of the research. Performed data collection, analysis, and preparation of the article. M.D.R., A.C.F.M. and L.T.T.: Collection, data analysis and analysis. L.A.M.: data collection and analysis and article preparation. C.G.B.C., G.C.B.A. and M.C.R.G.: Collection, data collection and analysis and article preparation. J.L.B.A.: Data collection and analysis and article preparation. A.S.S.: Founder of research, data collection, analysis and article preparation. All authors have approved the final version of the article. The authors declare that all data were generated in‐house and that no paper mill was used.

## Funding

This work was supported by the Coordination for the Improvement of Higher Education Personnel level (CAPES) and the National Council for Scientific and Technological Development (CNPq).

## Conflicts of Interest

All Author's (Ana Carla Lima de França; Mateus Duarte Ribeiro; Ana Carolina Freitas Meireles; Lydiane Tavares Toscano; Larissa Araújo Maia; Constantino Giovanni Braga Cartaxo; Gilvan da Cruz Barbosa Araújo; Maria da Conceição Rodrigues Gonçalves; José Luiz de Brito Alves; Alexandre Sérgio Silva) declare no conflicts of interest to disclose. This study was registered at ReBEC under number RBR‐96d3pvr.

## Data Availability

The data that support the findings of this study are available on request from the corresponding author. The data are not publicly available due to privacy or ethical restrictions.
